# Inhibition of Protein and Lipid Oxidation in Ready-to-Eat Chicken Patties by a *Spondias mombin* L. Bagasse Phenolic-Rich Extract

**DOI:** 10.3390/foods10061338

**Published:** 2021-06-10

**Authors:** Deocleciano C. de Santana Neto, Ângela M. T. M. Cordeiro, Bruno R. L. A. Meireles, Íris B. S. Araújo, Mario Estévez, Valquíria C. S. Ferreira, Fábio A. P. Silva

**Affiliations:** 1 Post-Graduate Program in Agro-Food Technology, Center for Human, Social and Agrarian Sciences, Federal University of Paraíba, Bananeiras 58220000, Paraíba, Brazil; deocleciano.cassiano7@gmail.com (D.C.d.S.N.); atribuzycordeiro@gmail.com (Â.M.T.M.C.); bruno_meireles7@hotmail.com (B.R.L.A.M.); iris.bsaraujo@gmail.com (Í.B.S.A.); valquiriacsf@gmail.com (V.C.S.F.); 2 IPROCAR Research Institute, University of Extremadura, 10003 Cáceres, Spain

**Keywords:** natural antioxidant, protein carbonylation, lipid peroxidation, fruit by-products, meat products

## Abstract

This study evaluated the impact of yellow mombin (*Spondias mombin* L.) bagasse extract (YMBE) on the color degradation, protein and lipid oxidation in ready-to-eat chicken patties during 15 days of refrigerated storage. Two formulations of chicken patties were developed: chicken patties control - PCON (without the antioxidant extract) and chicken patties with yellow mombin extract - PYME (with the antioxidant extract). The extract was effective in maintaining red color and inhibiting myoglobin degradation in the evaluated samples. The generation of lipid oxidation compounds during storage of the treated samples was delayed by 92.37% for peroxide index, 89.89% for conjugated dienes, 74.29% for tiobarbituric acid reactive substances (TBARs) and 92.55% for ρ-anisidine compared to the control samples. Moreover, the addition of YMBE inhibited the formation of carbonyl compounds during cold storage compared to the control samples. Extracts obtained from the yellow mombin bagasse act as a good natural antioxidant for ready-to-eat chicken patties inhibiting protein and lipid oxidative damage during cold storage, being a potential preservative to replace synthetic antioxidants in meat products.

## 1. Introduction

Chicken meat has desirable nutritional characteristics, as it has low lipid levels and is rich in polyunsaturated fatty acids [1,2]. However, chicken meat is susceptible to oxidative damage of lipids and proteins, which are the main causes of reduced shelf life and nutritional value in meat products [3,4,5,6,7].

Lipid oxidation leads to the generation of a wide range of degradation products that may be responsible for undesirable odors and flavors in meat and meat products [8]. These compounds can promote the onset of protein oxidation, during processing and storage, which leads to the loss of nutrients, such as essential amino acids (degradation of tryptophan, histidine, methionine and cysteine), resulting in decreased protein digestibility, color and texture degradation, and formation of potentially toxic compounds [4,9,10,11].

The use of synthetic antioxidants is one of the main strategies of the food industry to prevent the oxidation of lipids and proteins. However, the food industry has not measured efforts to reduce or replace the use of artificial additives in the preparation of processed products, given recent studies relating to the negative health effects of continuous consumption of synthetic compounds [6]. In this segment, the meat industry has invested in the search for natural ingredients that minimize oxidation reactions in meat products, thus increasing their shelf life.

In addition, the concern about sustainable and environmentally-friendly production is forcing the meat industry to continuously evolve. In this sense, the search for phenolic antioxidants from natural sources has received much attention. According to Helkar, Sahoo and Patil [12], by-products of plant origin include considerable amounts of vitamins, minerals and components of biological and technological interest. Many of these compounds can act as antioxidants.

Fruits of the genus *Spondias* are distributed worldwide in regions with tropical climates. The yellow mombin (*Spondias mombin* L.) is an exotic fruit of the family Anacardiaceae that is very widespread in America, Asia and Africa [13]. In Brazil, yellow mombin fruits are widely consumed fresh or in the form of pulp, especially in the North and Northeast, and they contain considerable amounts of carotenoids, phenolic compounds and other compounds with antioxidant potential [14]. In addition, the by-products of these tropical fruits have potential applicability for use as food additives (antimicrobials, antioxidant dyes, aromas and thickening agents) [15].

Despite extensive scientific data about the use of fruit and vegetable extracts with antioxidant potential in meat and meat products [1,2,5,16,17,18,19,20,21,22,23,24,25], elucidating the effects of some natural antioxidants obtained from tropical fruits in food products are still necessary. In fact, the influence of hydroethanolic extracts from *Spondias mombin* L. fruit bagasse on the oxidative stability of lipids and proteins in ready-for-consumption meat products have not been well elucidated. Given this knowledge gap, the aim of this study was to evaluate the effect of hydroethanolic extract prepared from yellow mombin bagasse (*Spondias mombin* L.) on lipid and protein oxidation parameters in ready-to-eat chicken patties.

## 2. Materials and Methods

### 2.1. Bagasse Collection and Preparation of the Yellow Mombin Bagasse Extract (YMBE)

The bagasse (husk, seed and residual pulp) from yellow mombin pulping (*Spondias mombin* L.) (approximately 4 kg) was collected in a fruit pulp agrobusiness located in the city of Esperança, Paraíba, Brazil. Bagasse was dried at 55 °C for 24 h in a forced air oven (SL–102/250, Solab, Piracicaba, SP, Brazil) [26]. The dehydrated bagasse was ground in a Willey mill (SL–31, Solab, Piracicaba, SP, Brazil), sieved (10 mesh) and stored at −80 °C until extraction.

The antioxidant extract of yellow mombin (YME) was obtained according to preliminary studies described in patent number BR102018067711-0 [27]. For the extraction, the dried bagasse was homogenized in 55% ethanol solution for 5 min and the homogenate was then incubated in a water bath during 35 min at 70 °C. Next, the mixture was centrifuged for 20 min at 8.960× *g* at 10 °C and the extract was filtered through qualitative filter paper (80 g·m^−2^). After extraction, ethanol was evaporated in a rotary evaporator (180 mbar and 45 °C), avoiding boiling of the extract. The final volume was adjusted to the initial content of the extract with distilled water. The extract was stored in an amber container and kept at −80 °C until processing of the chicken patty.

### 2.2. Total Phenolic Content and Phenolic Profile Analysis of the Extract

Total phenolic content was measured using the Folin Ciocalteau method. Results were expressed as mg gallic acid equivalents (GAE) per L extract.

The profile of phenolic compounds in the bagasse extract was determined using a high performance liquid chromatograph equipped with liquid chromatography (LC-20 AT) safety module (Shimadzu Corporation, Kyoto, Japan). Separation was performed on a C18 column (SUPELCOSIL™ LC-PAH, 250 mm × 4.6 ID, particle size 5 μm, Sigma-Aldrich, São Paulo, São Paulo, Brazil). Individual phenolic compounds were identified by spectroscopic Ultra Violet (UV) spectrum interpretation, retention time, and chromatographic comparison with authentic Sigma-Aldrich standards. The quantification was based on detection of peak areas using LabSolutions software version 5.42 SP4 (Shimadzu Corporation, Kyoto, Japan) versus pre-determined calibration curves. Calibration was performed by injecting the standards three times at five different concentrations (0.05, 0.1, 0.25, 0.5 and 1.0 mg·mL^−1^).

### 2.3. Experimental Design and Preparation of Chicken Patties

The patties were prepared using chicken thighs and drumsticks. A total of 40 chicken patties (approximately 80 g) were prepared in two separate processes following the general basic formulation: meat (70%), skin (10%), ice water (18%) and sodium chloride (2%). Two treatments were prepared: the control sample (PCON) was processed according to the base formulation, and the treated sample (PYME) was prepared with the addition of the extract of yellow mombin in the mixing step, replacing the water with an equivalent volume of extract (with a total of phenolic compounds of 200 mg of gallic acid per kg of chicken meat).

The skinless and deboned chicken thighs and drumsticks were ground in an industrial grinder (9I, WEG CAF, Rio Claro, Brazil) with a 6 mm disc and homogenized with the salt and ice water or yellow mombin bagasse extract (YMBE). The mixture was molded (approximately 80 g) into a patty shape (8 cm diameter and 1 cm thickness). The patties were then cooked on both sides for 10 min in an oven (NERO 90 L, Venax, Venâncio Aires, RS, Brazil) preheated to 170 °C. Next, the chicken patties were cooled to 25 °C and packaged in polyvinyl chloride film (oxygen permeability: ~17 cm^3^/m^2^ day atm, moisture permeability: <5 g·m^−2^ day) and expanded polystyrene trays, being stored under refrigeration in a BOD incubator (LT 320 TFP-I, Climatec, Bahia, Brazil) at 4 ± 1 °C for 15 days. The patties were collected every 5 days, being analyzed to measure instrumental color and oxidative stability.

### 2.4. Determination of Instrumental Color

The color parameters L* (luminosity), a* (red/green dimension), b* (yellow-blue dimension) and hue angle of the chicken patties were determined by direct reading at five different points of each sample using a Delta Vista d.0 colorimeter (Delta Vista Ltd., São Leopoldo, Brazil) using the CIELab system. To read the color coordinates, the following conditions were standardized: illuminant C, viewing angle 8°, and standard viewing angle of the observer 10°. The total colorimetric difference (ΔE) of chicken patty samples treated and not treated with antioxidant extract was determined and consistently calculated with samples at the beginning of storage, and was calculated according to Equation (1):

∆E = [(L*_0 day_ − L*_x days_)^2^ + (a*_0 day_ − a*_x days_)^2^ + (b*_0 day_ − b*_x days_)^2^]^1/2^ (1)

where x days correspond to the day of storage being compared.

### 2.5. Evaluation of the Oxidative Stability of Chicken Patties

#### 2.5.1. Peroxide Index

The peroxide index (PI) was determined according to the method described by Carvalho et al. [28] with modifications. The lipid extract obtained by Folch et al.’s [29] method was homogenized in 22.5 mL of glacial acetic acid, followed by the addition of 0.75 mL of saturated potassium iodide solution. The mixture was homogenized and kept in the dark for 1 min. Next, 22.5 mL of distilled water and 3 mL of starch solution (1%) were added as indicators, and the sample was titrated with 0.01 N sodium thiosulfate solution. The results were expressed in milliequivalents (meq) of active oxygen per kilo of lipid.

#### 2.5.2. Quantification of Conjugated Dienes

The conjugated dienes were determined according to the method proposed by the The International Union of Pure and Applied Chemistry (IUPAC) [30]. Aliquots of 0.5 g of the lipid (extracted from chicken patties by Folch et al. [29]) were dissolved in 25 mL of iso-octane and homogenized by vortexing. The dienes were quantified by measuring the absorbance at 234 nm, and the results were reported as absorbance $E_{1\mathrm{cm}}^{1\%}$, according to Equation (2):

(2) $$E_{1\mathrm{cm}}^{1\%}\left(234\right)=\left(\frac{A_{\lambda }}{C\times d}\right)$$

where *A_λ_* is the absorbance at 234 nm; *C* is the concentration of the lipid solution in iso-octane (g·100 mL^−1^), and *d* is the length of the cuvette.

#### 2.5.3. Thiobarbituric Acid Reactive Substances (TBARS Index)

The thiobarbituric acid reactive substances (TBARS) were extracted under acidic conditions and quantified using a spectrophotometer according to the method described by Ganhão et al. [31]. The content of malondialdehyde (MDA) was quantified using a standard curve of tetraethoxypropane (TEP), and the final result was expressed in mg of MDA/kg of sample. 

#### 2.5.4. *p*-Anisidine Value (*p*-AV)

The *p*-anisidine value was determined according to the method described by IUPAC [32]. The lipid fraction was obtained as described for conjugated dienes. Chicken patty lipid (0.5 g) was dissolved in 25 mL of n-hexane and the mixture was homogenized by vortexing. The absorbance was measured at 350 nm using a UV-Visible spectrophotometer (A_1_). Next, 1 mL of *p*-anisidine (2.5 mg·mL^−1^ in glacial acetic acid PA) was added to 5 mL of the lipid mixture, homogenized and kept in the dark for 10 min. The absorbance was then read at 350 nm (A_2_), and the *p*-anisidine value (*p*-AV) was calculated according to Equation (3):

(3) $$p-\mathrm{AV}=25\;\times \;\left(\frac{\left({1,2\;\times \;A}_{2}\right){-A}_{1})}{m}\right)$$

where m is the lipid mass used in the analysis.

#### 2.5.5. Quantification of Total Carbonyl Compounds

Protein oxidation of ready-to-eat chicken patties was evaluated by measuring the total carbonyl compound content, which was determined after derivatization of the sample with 2,4-dinitrophenylhydrazine (DNPH), according to the methodology proposed by Ganhão et al. [33]. The protein concentration of the samples was calculated by measuring the absorbance at 280 nm using a standard bovine serum albumin (BSA) curve. The number of carbonyl compounds was expressed in nmoles of carbonyl compounds per mg of protein using the molar extinction coefficient of hydrazones (21.0 nM^−1^ cm^−1^) at an absorbance of 370 nm.

### 2.6. Statistical Analysis

All the results were obtained in quadruplicate and analyzed by two-way ANOVA (analysis of variance) in order to evaluate the effect of natural extract, storage time, and their interaction using a completely randomized design (CRD) with a factorial scheme (2 treatments × 4 storage times). When the ANOVA revealed a significant effect (*p* < 0.05), the means were compared by the Tukey’s test at a significance of 5% using SAS^®^ software, version 9.2 (SAS, Cary, NC, USA). Differences detected by Tukey’s test were indicated by different letters.

## 3. Results and Discussion

### 3.1. Total Phenolic Content and Phenolic Profile of the YME

The analysis of total phenolic content in YME showed 1149.62 ± 1.4 mg GAE.L^−1^. The phenolic profile of YME identified 18 compounds. High concentrations of phenolic acids (652.84 ± 72.37 mg·100 g^−1^ dry matter) were observed, highlighting 2,5-dihydroxybenzoic acid (gentisic acid) (259.84 ± 22.29 mg·100 g^−1^ dm), salicylic (113.49 ± 12.48 mg·100 g^−1^ dm), 4-hydroxybenzoic acid (79.99 ± 10.44 mg·100 g^−1^ dm), and ellagic (46.93 ± 6.99 mg·100 g^−1^ dm). Furthermore, hydroxycinnamic acids, *p*-coumaric, ferulic, and caffeic, an important group of antioxidant substances, were found. A substantial amount of flavonoids (281.03 ± 58.85 mg·100 g^−1^ dm) was also achieved. Rutin (156.26 ± 23.38 mg·100 g^−1^ dm), catechin (66.90 ± 3.60 mg·100 g^−1^ dm), and myricetin (33.95 ± 6.55 mg·100 g^−1^ dm) were the flavonoids that stood out.

### 3.2. Effect of Refrigerated Storage on the Color of Chicken Patties

**Figure 1:**
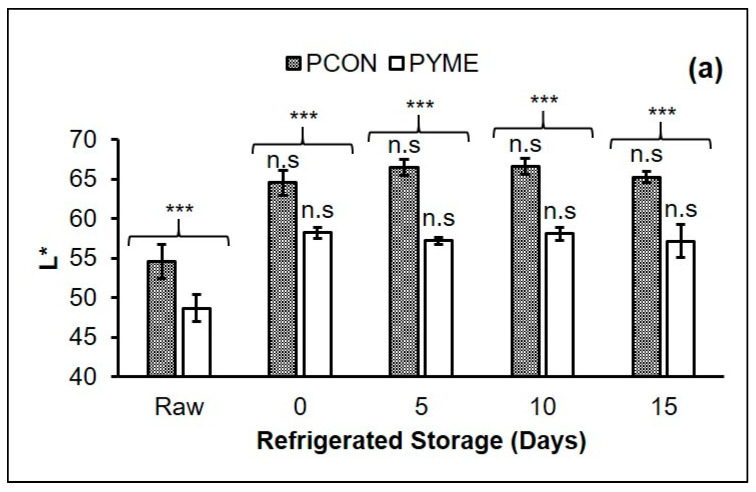

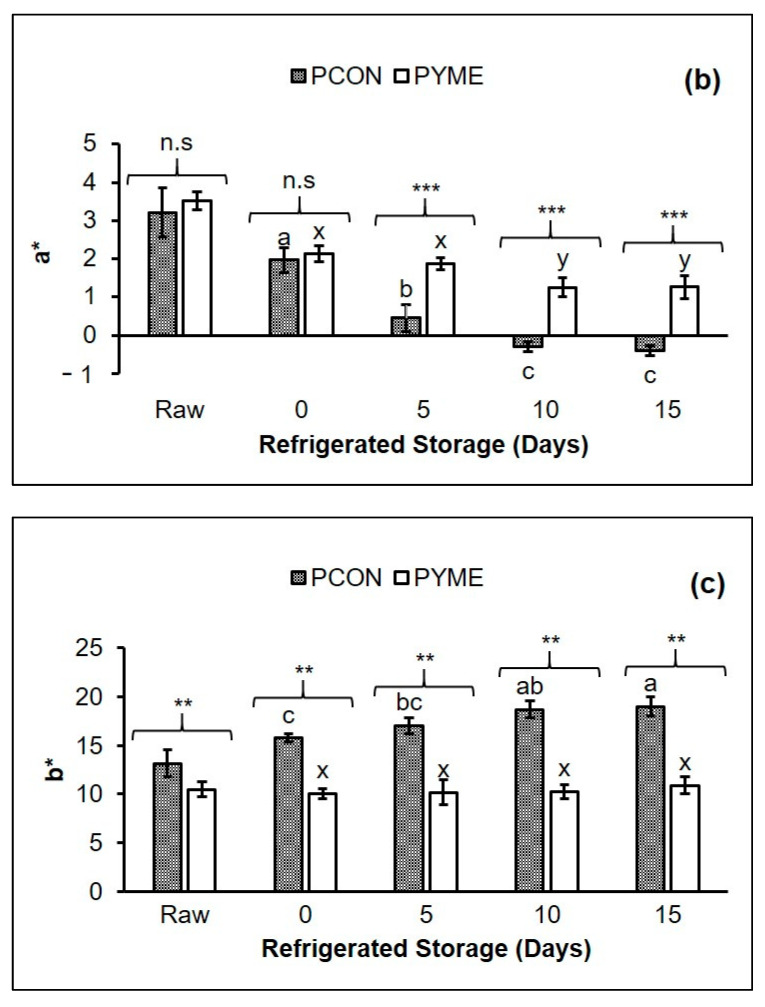

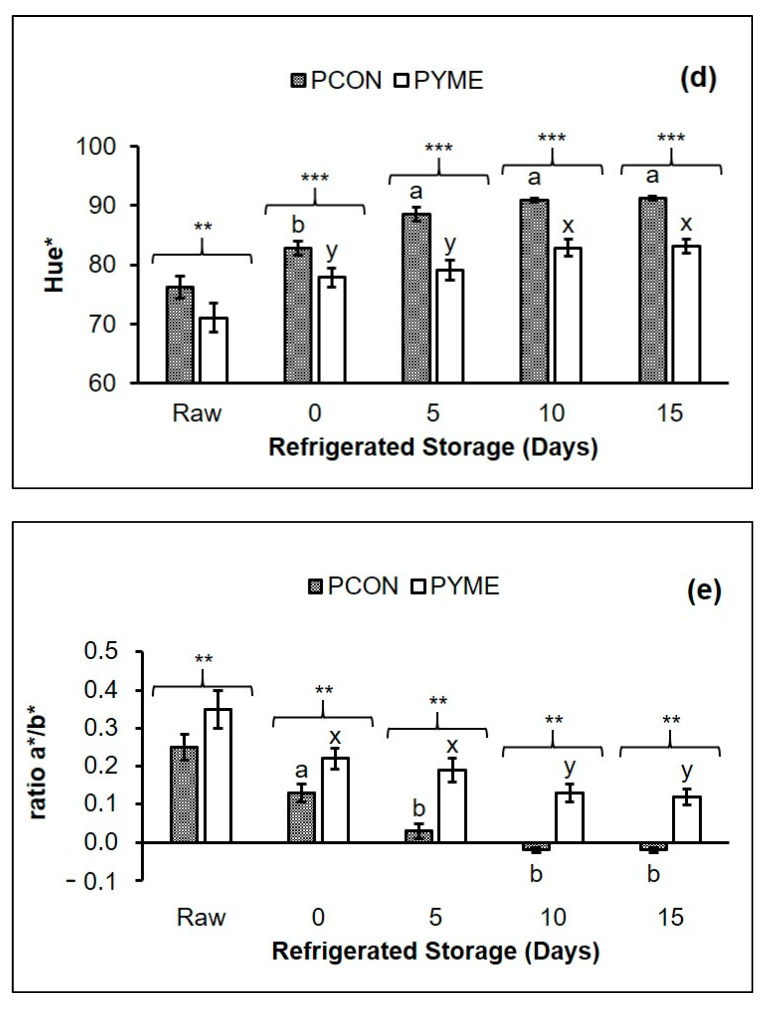

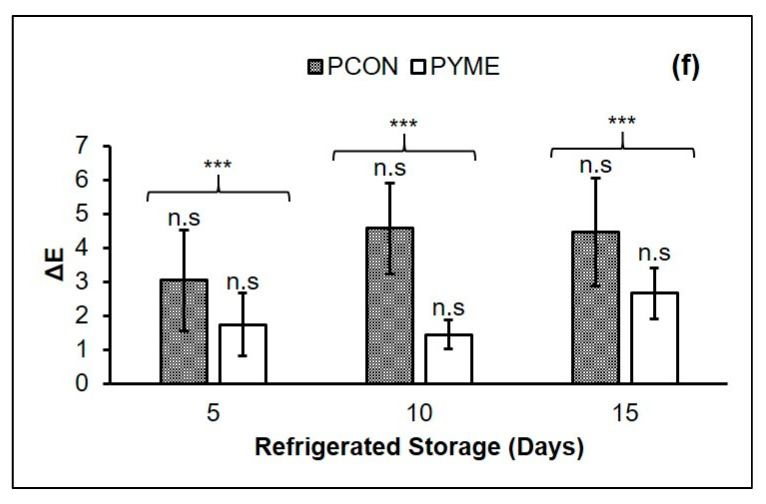
Effect of refrigerated storage (15 days at 4 ± 1 °C) on the color parameters of chicken patties without (PCON) and with the addition of yellow mombin extract (PYME). Results were expressed as mean ± standard deviation. L* (**a**), a* (**b**), b* (**c**), hue* (**d**) ratio a*/b* (**e**), and total colorimetric difference—ΔE (**f**). Footnotes: Different letters in the upper part of the bars (a–d means within the PCON samples and x–z means within the PYME samples) differ significantly (*p* < 0.05) between the days of refrigerated storage. The asterisks show significant differences between the samples on each day of refrigerated storage, n.s.: non-significant, **: *p* < 0.01 and ***: *p* < 0.001.

The effect of storage time and extract addition on the color parameters of the samples are shown in Figure 1. For lightness (L*), only an effect of the extract addition was observed (*p* < 0.05). PCON samples showed higher L* values (Figure 1a) than those of PYME treatment. Possibly, PYME samples were darker due to the interaction between YME and the patty muscle fibers. According to Devatkal et al. [34], the interaction between extracts of fruit by-products and salt in meat products may result in products with a darker color. Similar results were reported by Al-Juhaimi et al. [19] in beef patties with the addition of *Moringa oleifera* seeds.

The redness (a*) decreased (*p* < 0.05) during storage for both treatments (Figure 1b). However, red color of the treated sample faded very slowly compared to PCON. For treated and untreated samples, the redness reduction was only noted from the 10th (33.51%) and 5th day of storage (77.16%), respectively. This decrease in a* may be associated with the oxidation of myoglobin caused by reactive aldehydes generated in the lipid oxidation during storage [35]. The presence of red-orange pigments in the extract, such as carotenes, may also be responsible for the conservation of red color in the chicken patties during cold storage. Therefore, the incorporation of *Spondias mombin* L. extract had a protective effect on redness in the chicken patties. A similar behavior was observed by Munekata et al. [24] for cooked chicken patties with peanut skin extract.

Regarding the yellowness (b*), an interaction effect was observed. For the treated samples, storage did not cause changes (*p* > 0.05) in b* values, whereas in the control samples, b* changes were observed over the storage time (Figure 1c). This sharp increase in b* values for the control samples may be a consequence of myoglobin denaturation and metmyoglobin accumulation, with increased lipid and protein oxidation during storage [36].

The values obtained for hue* angle and the a*/b* ratio of the samples varied throughout the storage period, so the patties with antioxidant extract added showed greater stability (Figure 1d,e). According to Hernández et al. [37], the highest value for hue is related to the oxidation of myoglobin to generate metmyoglobin, giving the products a brown coloration. In fact, the PCON samples consistently showed higher hue* values than those of the treated samples due to degradation of the redness. A similar behavior was observed in raw chicken patties formulated with a combination of antioxidants during storage at 4 °C [2].

A decrease in the a*/b* ratio was noted during storage (Figure 1e) for both treatments. However, the addition of the YME was able to maintain the a*/b* ratio, suggesting a reduced myoglobin oxidation during storage. Faustman et al. [38] stated that the compounds present in antioxidant extracts interact with lipid oxidation products such as aldehydes and ketones, decreasing the oxidation of myoglobin. 

The greater conservation of red color, and consequently, the higher values for hue* and a*/b* ratio in the treated patties may be associated with the protective effect of phenolic compounds on myoglobin. The ability of phenolic compounds to protect against color changes in meat products can be attributed to free radical scavenging and the ability to chelate metals, such as iron ions, resulting in the formation of stable complexes with the heme and non-heme ions, and preventing the loss of color and inhibition of lipid oxidation [24,39]. In fact, in previous studies, the YME showed a high DPPH scavenging activity and an expressive ferric reducing antioxidant power (data not shown). The results of our experiment suggest that YME is effective for conserving the color of ready-to-eat chicken patties.

The PYME samples showed little variation in ΔE in relation to the beginning of storage (Figure 1f). According to Choi et al. [40], the difference in staining of samples with ΔE above 2.0 is noticeable to the human eye. In the Figure 1f, it was possible to observe that the application of YME in the ready-to-eat chicken patties kept the ΔE values lower than 2.0 until the 15th day of storage, while the PCON treatment showed ΔE values above 2.0 throughout the storage period.

### 3.3. Effect of Refrigerated Storage on Lipid and Protein Oxidation of Chicken Patties

**Figure 2:**
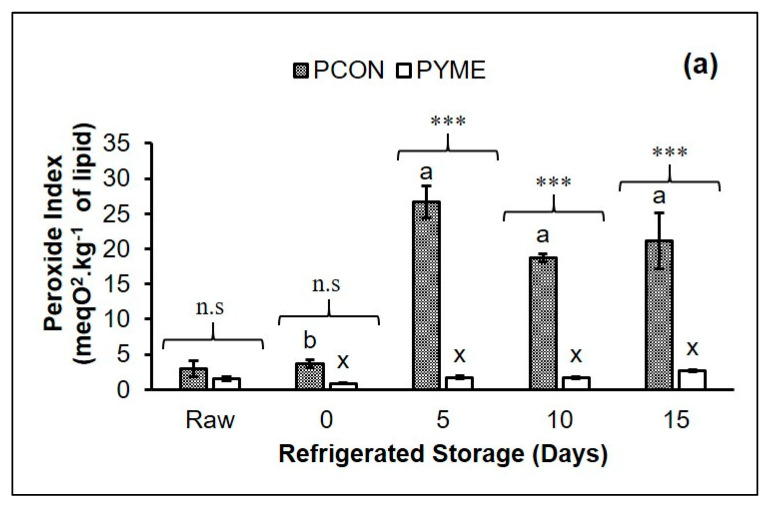

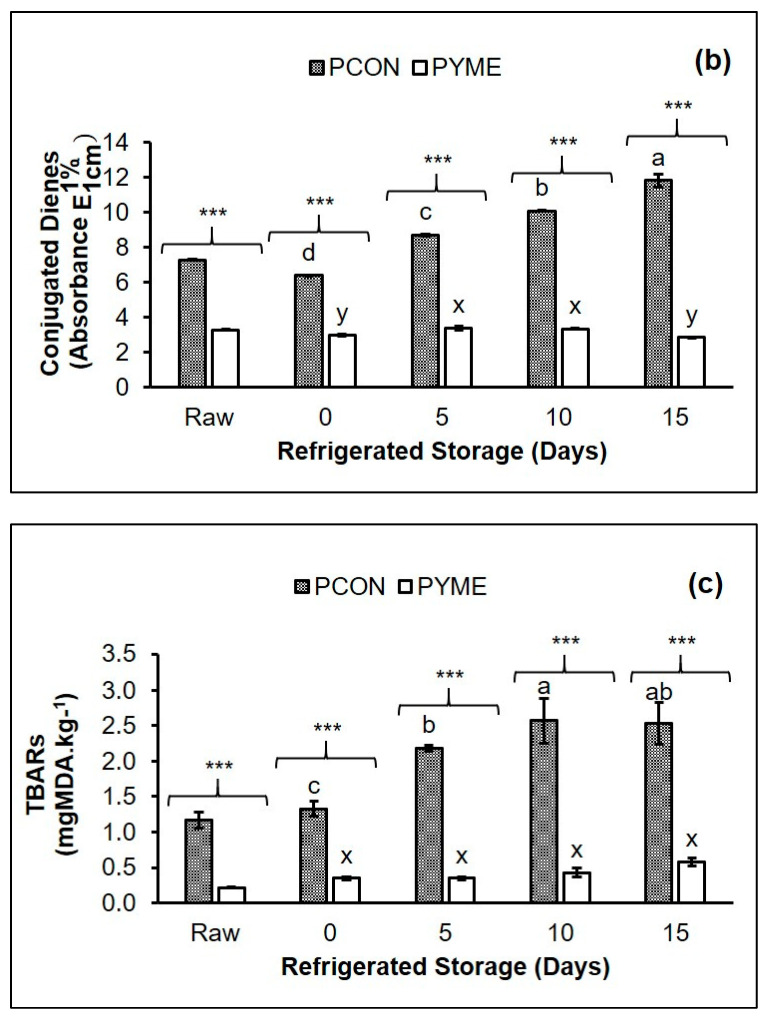

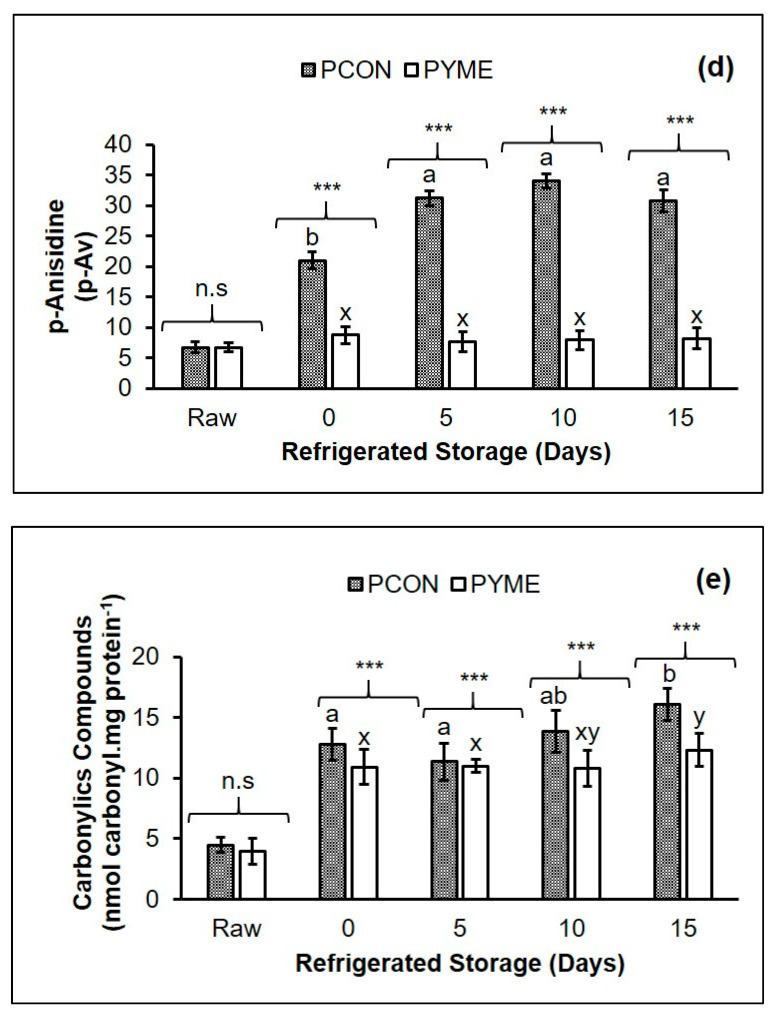
Effect of refrigerated storage (15 days at 4 ± 1 °C) on lipid and protein oxidation of chicken patty without (PCON) and with the addition of yellow mombin extract (PYME). Peroxide index (**a**), conjugated dienes (**b**), TBARS (**c**), *p*-anisidine (*p*-Av) (**d**), and formation of carbonyl compounds (**e**). Results were expressed as mean ± standard deviation. Footnotes: Different letters in the upper part of the bars (a–d means within the PCON samples and x–z means within the PYME samples) differ significantly (*p* < 0.05) between the days of refrigerated storage. The asterisks show significant differences between the samples on each day of refrigerated storage, n.s: non-significant, ***: *p* < 0.001.

Figure 2 illustrates the effect of the YME on the evolution of oxidation indices during refrigerated storage of ready-to-eat chicken patties. The incorporation of YME affected the lipid oxidation indices, and the PCON treatment showed the highest values.

For the primary lipid oxidation compounds, the application of YME retarded the generation of hydroperoxides (Figure 2a) and conjugated dienes (Figure 2b) during refrigerated storage compared with PCON. A hydroperoxide generation peak was observed in PCON, followed by a reduction after the 5th day of storage. This decrease may be related to the consumption of hydroperoxides for the formation of secondary compounds of lipid oxidation, as observed in other reports for samples of cooked meats [2,23]. The hydroperoxide generation of PYME samples remained low (<3.0 meqO_2_.kg^−1^ of lipid) during 15 days of cold storage, suggesting that the addition of YME inhibited hydroperoxide generation in the samples during cooking and refrigerated storage. The results observed in our experiment for the PYME samples were lower than those reported by Ergezer and Serdaroğlu [21] in raw beef patties incorporated with artichoke by-product extracts.

A greater formation of conjugated dienes (CD) was observed during storage in the control samples. For PYME treatment, a slight increase in CD was noted between the 5th and 10th day of storage, followed by a decrease. This decrease in CD content may result from the degradation of conjugated hydroperoxides and the formation of secondary products of lipid oxidation [21]. Our results are consistent with that reported by Hwang et al.’s [2] study of the antioxidant effect of *Artemisia princeps* Pamp extract alone and combined with ascorbic acid in raw chicken patties during refrigerated storage.

Results of the secondary lipid oxidation compounds are described in Figure 2c (TBARS) and Figure 2d (*p*-Av). The effect of the antioxidant extract, storage time, and interaction in TBARS and *p*-Av formation of evaluated samples was observed. 

A reduced TBARS’ value was found in treated samples immediately after processing. For both treatments, cooking did not increase the formation of TBARS. During storage, TBARS’ values ranged from 1.33 to 2.57 mg MDA.kg^−1^ in control samples and from 0.35 to 0.58 mg of MDA.kg^−1^ in treated chicken patties. These results demonstrated an inhibition rate of TBARS’ generation of 74.29%. Furthermore, the inhibitory effect of YME on TBARS’ formation was clearly evident because no significant increase in TBARS was observed even after cooking and during the entire refrigerated storage of the PYME samples.

The lower TBARS’ values in the treated samples can be ascribed to the phenolic compounds’ activity [24,39]. In fact, YME showed high concentration of gentisic acid and rutin (already reported), which were reported as effective antioxidant compounds against lipid oxidation [17,20]. Joshi et al. [41] proposed that the antioxidant properties of gentisic acid are exerted by its phenoxyl group, which acts as scavenging lipid radicals. The TBARS’ results of this experiment were similar to those reported by Munekata et al. [24], who observed a decrease in TBARS’ generation in chicken meat supplemented with peanut skin extract.

After cooking, the patties showed an increase of 30.58% in *p*-Av for the PYME sample (from 6.67 to 8.71), while the PCON sample showed an increase of 214% in relation to the raw sample (from 6.67 to 20.99). The synthesis of aldehyde compounds in treated samples were lower than PCON treatment. This reduction shows that YME exerts an antioxidant effect, inhibiting oxidation, which is in agreement with the previously described measures. These compounds (aldehydes, mainly 2-alkenyl and 2,4-alkadienals) originate from the decomposition of hydroperoxides, resulting in the development of unpleasant flavors and odors in lipids, indicating an advanced stage of lipid oxidation [1,21].

A constant increase in *p*-Av values of PCON samples were observed during refrigerated storage, indicating an advanced oxidation stage from the 5th day of storage, culminating in the peak hydroperoxide generation and accumulation of aldehydes in not- treated chicken patties. A similar behavior was perceived by Ergezer and Serdaroğlu [21] and Cagdas and Kumcuoglu [1] in meat products under refrigerated and frozen storage supplemented with artichoke and grape seed extracts, respectively.

The evolution of carbonyl compounds in patty samples can be observed in Figure 2e. Significant differences (*p* < 0.05) in the carbonyl compounds were perceived between samples after cooking as well as for refrigerated storage. The addition of YME resulted in reduced carbonyl compound generation (10.78 to 12.32 nmol of carbonyls/mg of protein) in relation to the control samples (11.34 to 16.09 nmol of carbonyls/mg of protein), indicating an inhibition in the formation of total carbonyl compounds by 28.01%. Furthermore, the carbonyl content of PYME samples showed no variation (*p* > 0.05) between the beginning and the tenth day of storage, demonstrating a storage effect only after 10 days.

Protein oxidation can be induced by the presence of lipid oxidation compounds when hydroperoxides are decomposed to form carbonyls and other substances (aldehydes), which easily oxidize protein constituents, including thiols and amino acids, leading to the generation of carbonyl compounds [42]. The formation of carbonyls is considered a general indicator to evaluate the level of protein oxidation in meat and meat derivatives, and cooking was observed to be responsible for a greater increase in carbonyl compound content in this experiment.

The lower total carbonyl content in the treated samples could be attributed to the phenolic acids and flavonoids identified in the YME, such as gallic acid, rutin, and catechin, which are effective in inhibiting the formation of total protein carbonyl compounds [43]. Rutin, for example, can directly scavenge reactive oxygen species (ROS) because of its chemical structure [44]. Studies using plant extracts containing phenolic compounds to inhibit/decrease the oxidation rate of lipids and proteins have been reported in the literature [33]. Pasukamonset et al. [16] reported lower values for total carbonyl compounds for boiled pork patties supplemented with *Clitoria ternatea* extract during 12 days of storage; this approach was found to be as effective as the use of BHT (butylated hydroxytoluene) in inhibiting protein oxidation.

## 4. Conclusions

Considering the obtained results, the incorporation of yellow mombin extract on the formulation of ready-to-eat chicken patties positively affects the rate of lipid and protein oxidation. Based on these results, the antioxidant extract of yellow mombin bagasse can be considered an effective natural antioxidant, capable of inhibiting lipid oxidation and carbonylation of proteins, as well as conserving the color of ready-to-eat chicken patties during refrigerated storage. As a by-product of juice processing, aqueous extract of yellow mombin bagasse seems to be a good and cheap alternative to replace synthetic antioxidants in meat products. However, additional studies are needed to evaluate the effect of yellow mombin bagasse extract on sensory and microbiological properties of meat products, in addition to evaluating the viability of alternative delivery systems of the antioxidant compounds present in the extract, such as active packaging.

## Data Availability

Not applicable.
